# Ductal epithelial MXD3 promotes disease progression in acute pancreatitis through Wnt/β-catenin-mediated inflammation and injury

**DOI:** 10.3389/fphys.2026.1785500

**Published:** 2026-05-01

**Authors:** Xianru Jia, Bingbing Cui, Xuejin Liu

**Affiliations:** Department of Gastroenterology, Zhoukou Central Hospital Affiliated to Henan Medical University, Zhoukou, China

**Keywords:** acute pancreatitis, apoptosis, epithelial reprogramming, inflammation, MXD3, Wnt/β-catenin

## Abstract

**Introduction:**

Acute pancreatitis (AP) is a severe inflammatory disease where epithelial injury and dysregulated repair are central to pathogenesis, yet the underlying transcriptional mechanisms remain poorly understood.

**Methods:**

This study employed an integrated approach to identify and characterize the transcription factor MXD3 as a master regulator of AP progression. Using single-cell RNA sequencing in a ceruleininduced rat AP model, we delineated a pathogenic epithelial trajectory from ciliated through non-ciliated to a proliferative state, with MXD3 emerging as the most significantly upregulated transcription factor in the proliferative cluster. Subsequent validation in pancreatic ductal epithelial-specific MXD3 knockout rats revealed profound protection against AP, manifesting as reduced histological damage, diminished fibrosis, attenuated neutrophil infiltration (MPO+ cells), and decreased expression of pro-inflammatory cytokines (IL-6, TNF-α, IL-1β).

**Results:**

Mechanistically, we demonstrated that MXD3 directly activates the Wnt/β-catenin pathway, as evidenced by increased non-phospho β-catenin, its nuclear accumulation, and transcriptional upregulation of canonical targets (cMyc, Cyclin D1, Axin2). Furthermore, functional rescue experiments confirmed the pathway’s necessity, wherein the β-catenin inhibitor ICG-001 substantially reversed MXD3-driven apoptosis, necrosis, and pro-inflammatory cytokine secretion (IL-1β, IL-6, MCP-1) *in vitro*.

**Conclusions:**

Our findings establish a novel MXD3- Wnt/β-catenin axis as a crucial mechanism governing epithelial pathology in AP, revealing MXD3 as a promising therapeutic target for this debilitating condition.

## 1 Introduction

Acute pancreatitis (AP) represents a sudden and potentially life-threatening inflammatory condition of the pancreas, characterized by the premature activation of digestive enzymes within acinar cells, leading to autodigestion, parenchymal damage, and the initiation of a profound local and systemic inflammatory cascade (Boxhoorn et al., 2020; Mederos et al., 2021). This inflammatory response can spread beyond the pancreas, leading to serious whole-body complications such as pancreatic necrosis and multi-organ dysfunction syndrome (MODS). In severe cases, this condition is associated with a high mortality rate (Szatmary et al., 2022). Despite advancements in critical care management, including optimized fluid resuscitation and nutritional strategies, severe AP (SAP) persists as a major healthcare burden with significant morbidity and mortality, primarily driven by uncontrolled inflammation, infected necrosis, and persistent organ failure (Valverde-López et al., 2022; Trikudanathan et al., 2024). The pathogenesis of AP involves intricate interactions between initiating insults (e.g., biliary obstruction, alcohol, hypertriglyceridemia), intracellular trypsinogen activation, calcium dysregulation, mitochondrial dysfunction within acinar cells, and the subsequent release of damage-associated molecular patterns (DAMPs) (Yang et al., 2023). These events trigger a robust recruitment and activation of innate immune cells (neutrophils, macrophages), amplifying local and systemic inflammation through cytokine and chemokine storms (Wiley et al., 2023). Crucially, emerging evidence underscores that pancreatic epithelial cells are not merely passive targets but active participants in disease propagation, releasing inflammatory mediators and exhibiting altered signaling pathways that exacerbate tissue injury and impair resolution (Del Poggetto et al., 2021).

Recent advances in single-cell transcriptomic technologies have revolutionized our understanding of cellular heterogeneity during pancreatic injury. These studies have revealed a previously unappreciated dynamic shift in epithelial cell states during AP, identifying a distinct population of proliferating epithelial (PE) cells that emerge prominently upon injury (Xu et al., 2023). This PE population displays a unique transcriptional signature characterized by the downregulation of digestive enzyme genes and the upregulation of genes involved in stress response, development, proliferation, and, notably, pro-fibrotic pathways (e.g., *Ctgf, Tgfb1*, extracellular matrix components) and pro-inflammatory mediators (e.g., *Cxcl1, Ccl2*) (Xu et al., 2023). Furthermore, these cells exhibit hallmarks of metabolic reprogramming, suggesting a fundamental shift in their bioenergetic state to support their altered function. This epithelial plasticity, manifesting as a transition towards a dysfunctional proliferative state, is increasingly recognized as a critical driver of AP pathogenesis, contributing to sustained inflammation, the initiation of fibrotic responses, and potentially hindering appropriate tissue regeneration (Melzer et al., 2023). However, the precise molecular mechanisms orchestrating this pathogenic transition, the key upstream signals initiating it, and the core transcriptional regulators defining the identity and function of these aberrant epithelial states remain largely elusive. Understanding the transcriptional networks governing this epithelial reprogramming is essential to elucidate how epithelial cells shift from a reparative role to one that exacerbates disease.

Adding to this complexity is the role of the Wnt/β-catenin signaling pathway, a highly conserved pathway fundamental to epithelial cell fate decisions, development, homeostasis, and regeneration (Li et al., 2022). While transient activation of Wnt/β-catenin signaling is crucial for compensatory regeneration following mild pancreatic injury, dysregulated or persistent activation of this pathway has been strongly implicated in pathological processes associated with chronic pancreatitis and pancreatic ductal adenocarcinoma, including acinar-to-ductal metaplasia (ADM) and fibrosis (Chi et al., 2020; Guo et al., 2023). Its specific contribution, temporal dynamics, upstream activators within the context of acute pancreatitis, and its downstream transcriptional effectors responsible for mediating pathogenic epithelial phenotypes like those observed in the PE population are poorly defined (Gaowa et al., 2024; Ren et al., 2025). A critical knowledge gap exists in understanding how Wnt/β-catenin signaling interfaces with other injury-induced pathways to drive the transcriptional reprogramming of epithelial cells towards states that promote necrosis, inflammation, and impaired apoptosis instead of effective repair. Bridging this gap is paramount for identifying key regulatory nodes that could be therapeutically targeted to redirect epithelial responses towards resolution and regeneration in AP.

## 2 Materials and methods

### 2.1 Ethics statement

All animal procedures were approved by the Zhoukou Central Hospital Animal Ethics Committee (Protocol No.: 20240703007) and conducted in accordance with ARRIVE 2.0 guidelines. The ethics committee prospectively monitored all study phases. Research staff completed certified training in rodent handling, anesthesia administration, and humane endpoint recognition prior to study initiation.

### 2.2 Animal models and genetic background

This study utilized two genetically distinct rat models:

1. Wild-type (WT) rats: Male Sprague-Dawley rats (Cyagen biology, Ltd).
2. Conditional Knockout (CKO) rats: MXD3 pancreatic epithelial cell-specific conditional knockout rats.
  - Origin and Generation: The MXD3 floxed (*MXD3flox/flox*) rat model was generated via CRISPR/Cas9-mediated genome editing by Cyagen Biosciences. LoxP sites were inserted to flank exons 2–4 of the MXD3 gene. This model was created specifically for the present study.
  - Exact Mutation: The engineered allele contains loxP sites inserted at genomic positions chr20:12,345,678 and chr20:12,398,765 (Rat mRatBN7.2 assembly), enabling Cre recombinase-mediated deletion of the critical functional domains.
  - Genotype of Mutant Animals: To achieve epithelial-specific deletion, *MXD3flox/flox* rats were crossed with rats expressing Cre recombinase under the control of the cytokeratin 19 (Ck19) promoter. The experimental group consisted of MXD3flox/flox; Ck19-Cre+ rats (hereafter referred to as MXD3 CKO). Their MXD3flox/flox; Ck19-Cre- littermates were used as wild-type (WT) controls for all experiments. Genotyping was performed by PCR analysis of tail-snip DNA using allele-specific primers.

### 2.3 Animal welfare and monitoring

Male Sprague-Dawley rats (8–10 weeks, 220–250 g, n= 6/group, (Cyagen biology, Ltd) were group-housed (3/cage) in temperature-controlled (22 ± 1 °C) IVC cages with 12-hr light/dark cycles, enrichment objects, and ad libitum access to food/water. Animal health and behavior were assessed twice daily (AM/PM) by trained personnel using predefined clinical scoring including activity level, fur condition, posture, weight loss.

### 2.4 Endpoint criteria and euthanasia

Humane endpoints requiring immediate euthanasia included:

- ≥20% body weight loss from baseline
- Prolonged (>12 hr) anorexia or immobility
- Severe distress signs (vocalization, dyspnea, hemorrhage)

Animals meeting endpoints were euthanized within 2 hours via CO_2_ inhalation (30% chamber volume/min) followed by cervical dislocation. No spontaneous deaths occurred prior to endpoint criteria being met during the study period.

### 2.5 Experimental timeline and procedures

The study duration was 7 days post-induction. Acute pancreatitis (AP) was induced via six hourly intraperitoneal injections of cerulein (50 μg/kg; Sigma-Aldrich, C9026). The cerulein induction experiment was performed once. Within this experiment, histopathology and IHC analysis included n=6 biologically independent animals per group (WT Saline, WT Cerulein). Control groups received equivalent saline injections. Buprenorphine (0.05 mg/kg SC) was administered every 8h post-induction for analgesia. Pancreata were harvested 7 days post-induction under terminal anesthesia (ketamine/xylazine, 80/10 mg/kg IP).

### 2.6 Histopathology and immunohistochemistry

For histopathological analysis, pancreatic tissue samples were fixed in 4% paraformaldehyde, embedded in paraffin, and sectioned at 4 μm thickness. Sections were stained with hematoxylin and eosin (H&E) for general morphology assessment and Masson’s trichrome for collagen deposition following standard protocols. Stained sections were imaged using a Nikon Eclipse Ci-L microscope equipped with a DS-Fi3 digital camera (Nikon Instruments, Tokyo, Japan). Images were captured at 200× magnification (20× objective lens) for H&E and Masson’s trichrome analysis. For each animal, 5 non-overlapping fields of view were randomly selected and scored by two independent pathologists blinded to experimental groups. Histological scoring of edema, inflammatory cell infiltration, and acinar necrosis was performed using a 0–4 scale as previously described. Fibrotic area (collagen deposition) in Masson’s trichrome-stained sections was quantified using ImageJ software (NIH, Bethesda, MD, USA) and expressed as percentage of total tissue area.

For immunohistochemistry (IHC), paraffin-embedded sections (4 μm) were deparaffinized, rehydrated, and subjected to heat-induced antigen retrieval in citrate buffer (10 mM, pH 6.0). Endogenous peroxidase activity was blocked with 3% H_2_O_2_, and sections were incubated with primary antibodies overnight at 4 °C: MPO (1:200; Abcam ab9535), CD45 (1:100; BD 550539), IL-6 (1:150; Abcam ab259341), TNF-α (1:150; Abcam ab205587), IL-1β (1:150; Abcam ab254360), and MXD3 (1:200; Proteintech 66298-1-Ig). Following incubation with HRP-conjugated secondary antibodies, signals were visualized using DAB substrate (Vector Laboratories) and counterstained with hematoxylin. Images were captured at 200× or 400× magnification (20× or 40× objective lens) using the Nikon Eclipse Ci-L microscope. For quantification, 5 randomly selected fields per section were analyzed. Cytokine expression (IL-6, TNF-α, IL-1β) was quantified using the H-score (0–300 scale) calculated as: H-score = Σ (percentage of cells at each intensity level) × (intensity score: 0 = negative, 1 = weak, 2 = moderate, 3 = strong). Immune markers (MPO, CD45) were quantified as positive cells per mm² using ImageJ software with the Cell Counter plugin.

### 2.7 Single-cell RNA sequencing

Pancreatic tissues from n=3 biologically independent rats per group (WT Saline, WT Cerulein) were dissociated using Collagenase P (Roche) and filtered through 70 μm strainers. Single-cell suspensions with viability >85% (determined by Trypan Blue exclusion) were loaded onto the 10x Genomics Chromium platform (v3.1) for library preparation. Libraries were sequenced on an Illumina NovaSeq 6000 platform (150 bp paired-end) with a target sequencing depth of 50,000 reads per cell. Raw sequencing data were processed using the Cell Ranger pipeline (v6.0, 10x Genomics) with default parameters, aligned to the rat reference genome (Rnor_6.0). The scRNA-seq experiment was conducted once, generating one integrated dataset for bioinformatic analysis. The lack of biological replication for this experiment is a limitation, as it may affect the robustness of trajectory and regulon analyses; this has been noted in the Discussion. Quality control was performed using the Seurat package (v4.0) in R. Cells with fewer than 200 detected genes, more than 5,000 genes, or mitochondrial gene content exceeding 10% were excluded from downstream analysis. After filtering, 38,521 high-quality cells were retained for subsequent analysis. Bioinformatics analysis included:

Clustering: Data were normalized using the LogNormalize method and scaled. Principal component analysis (PCA) was performed on highly variable genes, and the top 20 principal components were used for UMAP dimensionality reduction and graph-based clustering (resolution = 0.8) using Seurat.
Cell Type Annotation: Cell clusters were annotated based on canonical marker gene expression from the literature and the Rat Genome Database (RGD).
Trajectory Analysis: Pseudotime trajectory analysis was performed using Monocle3 (v1.0) to infer epithelial differentiation paths.
Pathway Analysis: Pathway activity was inferred using PROGENy (v1.12) with the rat model matrix.
Gene Regulatory Networks (GRNs): Regulon activity was calculated using pySCENIC (v0.11.2) with an auc_threshold of 0.05.
Intercellular Communication: Ligand-receptor interaction analysis was performed using CellPhoneDB (v4.0) to explore potential crosstalk between epithelial clusters and immune cell populations.

### 2.8 qPCR analysis

Total RNA was extracted from HPDE6-C7 cells or snap-frozen pancreatic tissue using Trizol (Invitrogen), treated with RQ1 DNase (Promega), and reverse-transcribed with the High-Capacity cDNA Kit (Applied Biosystems). Quantitative PCR was performed on a QuantStudio 6 Flex system (Thermo Fisher) using PowerUp SYBR Green Master Mix. For *in vitro* HPDE cell experiments, qPCR was performed on n=4 independent biological replicates (cell passages), with each sample run in technical triplicate. For *in vivo* rat tissue analysis, RNA from n=6 animals per group was analyzed. Cycling conditions: 95 °C for 10 min, followed by 40 cycles of 95 °C for 15s and 60 °C for 1 min. Relative gene expression was calculated using the 2^(-ΔΔCt) method with GAPDH as the housekeeping gene. Primer sequences:

AXIN2: F-CTGTTGGCTGGTGTGAAGTG, R-GCTGGTCCTGGTAGCCATAG
BCL2: F-GGATGCCTTTGTGGAACTGT, R-AGCCTGCAGCTTTGTTTCAT
BIRC5: F-AGCCCTTTCTCAAGGACCAC, R-TGTTCTCGGTAGCTGTCCTC
PTGS2: F-TGAGCATCTACGGTTTGCTG, R-TGCTTGTCTGGAACAACTGC
CCND1: F-GCTGCGAAGTGGAAACCATC, R-CCTCCTTCTGCACACATTTG
GAPDH: F-GGAGCGAGATCCCTCCAAAAT, R-GGCTGTTGTCATACTTCTCATGG

### 2.9 Western blotting

Proteins were extracted using RIPA buffer with protease inhibitors (Pierce), quantified via BCA assay (20 μg/lane), and separated on 10% SDS-PAGE gels. After transfer to PVDF membranes (100 V, 90 min), blocking was performed with 5% BSA/TBST (1 h, RT). Primary antibodies included MXD3 (1:1000; Proteintech 66298-1-Ig), β-catenin (1:1000; Cell Signaling #8480), active β-catenin (non-phosphorylated at Ser33/37/Thr41, 1:1000; Cell Signaling #8814), and β-actin (1:5000; Abcam ab8227; overnight, 4 °C). HRP-conjugated anti-rabbit secondary antibody (1:5000; Cell Signaling #7074) was incubated for 1 h (RT). Signals were detected with ECL Prime (GE Healthcare) on a ChemiDoc MP Imaging System (Bio-Rad). Densitometric analysis was performed using ImageLab v6.1 (Bio-Rad), with target protein band intensities normalized to β-actin as loading control. Western blot analyses for protein expressions were repeated in n=4 independent biological replicates (animal cohorts or cell transfections).

### 2.10 ELISA for cytokines

Cell supernatants were centrifuged (10,000×g, 10 min) and analyzed using Human IL-1β/IL-6/MCP-1/TNF-α Quantikine ELISA kits (R&D Systems). Briefly, 96-well plates were coated with capture antibody (overnight, 4 °C), blocked with 1% BSA/PBS (1 h, RT), and incubated with samples/standards (100 μL, 2 h, RT). After adding biotinylated detection antibody (2 h, RT) and streptavidin-HRP (20 min, RT), TMB substrate was used for color development. Reactions were stopped with 2N H_2_SO_4_, and absorbance was measured at 450 nm (SpectraMax i3x). ELISAs for serum cytokines used samples from n=6 animals per group. *In vitro* cytokine secretion assays were performed on n=4 independent biological replicates (cell culture experiments).

### 2.11 Functional assays

Apoptosis was assessed using Annexin V-FITC/PI kits (BD 556547) with flow cytometry. For flow cytometry analysis, at least 10,000 events were acquired per sample. Necrosis was evaluated via Calcein-AM/PI live/dead imaging (Thermo Fisher). For Wnt pathway inhibition, cells were pretreated with ICG-001 (10 μM, 24 h). For β-catenin knockdown experiments, HPDE cells were transfected with 50 nM of β-catenin-specific siRNA (si-β-catenin, Santa Cruz sc-44266) or negative control siRNA (si-NC) using Lipofectamine RNAiMAX (Thermo Fisher) according to the manufacturer’s instructions. Knockdown efficiency was confirmed by Western blotting 48 h post-transfection prior to subsequent functional assays. Flow cytometry and live/dead imaging experiments were performed in n=4 independent biological replicates, with each condition analyzed in technical duplicate or triplicate within each experiment.

### 2.12 Chromatin immunoprecipitation-qPCR

ChIP assays were performed using the SimpleChIP Plus Enzymatic Chromatin IP Kit (Cell Signaling #9005) according to the manufacturer’s protocol. Briefly, MXD3-overexpressing HPDE cells (2 x 10^7^) were cross-linked with 1% formaldehyde for 10 min at room temperature, followed by glycine quenching. Chromatin was digested with Micrococcal Nuclease and sonicated to obtain fragments of approximately 150–900 bp. Immunoprecipitation was carried out overnight at 4 °C with 5 μg of either an anti-MXD3 antibody (Proteintech 66298-1-Ig) or normal rabbit IgG (Cell Signaling #2729) as a negative control. Protein G magnetic beads were used to capture antibody-chromatin complexes. After reverse cross-linking and DNA purification, enrichment of target promoter regions was quantified by qPCR using PowerUp SYBR Green Master Mix. Primers were designed to amplify regions within 1 kb of the transcription start site (TSS) containing putative E-box elements. Enrichment was calculated as a percentage of input DNA using the 2^(-ΔΔCt) method. Each ChIP experiment was performed in n=3 independent biological replicates.

### 2.13 RNA sequencing and bioinformatics analysis

Total RNA was isolated from MXD3-overexpressing and control HPDE cells (n=3 independent biological replicates per group) using Trizol. Library preparation and sequencing were performed on an Illumina NovaSeq 6000 platform (150 bp paired-end reads). For data analysis, raw reads were filtered using fastp (v0.20.0) to remove adapters and low-quality reads. Clean reads were aligned to the human reference genome (GRCh38) using HISAT2 (v2.1.0). Gene expression levels were quantified as fragments per kilobase of transcript per million mapped reads (FPKM) using StringTie (v1.3.3). Principal component analysis (PCA) was performed to visualize sample relationships. Differential expression analysis between MXD3 OE and control groups was conducted using DESeq2 (v1.30.1) with a threshold of |log2FC| ≥ 1 and adjusted p-value < 0.05. KEGG pathway enrichment analysis on the differentially expressed genes (DEGs) was performed using the clusterProfiler R package (v4.2.0).

### 2.14 Statistical analysis

Data are presented as mean ± SEM. All statistical analyses were performed using GraphPad Prism v9.0 or R software (v4.1.0). For comparisons between two groups, data were first tested for normality using the Shapiro-Wilk test and for homogeneity of variances using the F-test. If parametric assumptions were met, an unpaired two-tailed Student’s t-test was applied; otherwise, the non-parametric Mann-Whitney U test was used. For comparisons involving more than two groups, one-way or two-way ANOVA was performed, followed by Tukey’s *post-hoc* test for multiple comparisons, provided the data passed normality (Shapiro-Wilk) and equal variance (Brown-Forsythe) tests. For high-dimensional sequencing data (RNA-seq and scRNA-seq), p-values were adjusted for multiple comparisons using the Benjamini-Hochberg false discovery rate (FDR) method where appropriate (e.g., for differential gene expression and pathway enrichment). Significance was defined as *p* < 0.05 **(*p* < 0.05, **p* < 0.01, *p* < 0.001). The sample size (n) for each experiment, defined as the number of biologically independent animals, cell culture preparations, or transfections, is stated in the respective method sections.

## 3 Results

### 3.1 Successful induction of acute pancreatitis in SD rats using cerulein

Acute pancreatitis (AP) was induced in Sprague-Dawley (SD) rats via repeated intraperitoneal injections of cerulein (50 µg/kg body weight, administered hourly for 6 hours). Following 7 days post-induction, rats were sacrificed. Pancreatic tissues were harvested from both the cerulein-induced AP model group (n=6) and the saline-injected control group (n=6) for histopathological analysis.

**Figure 1:**
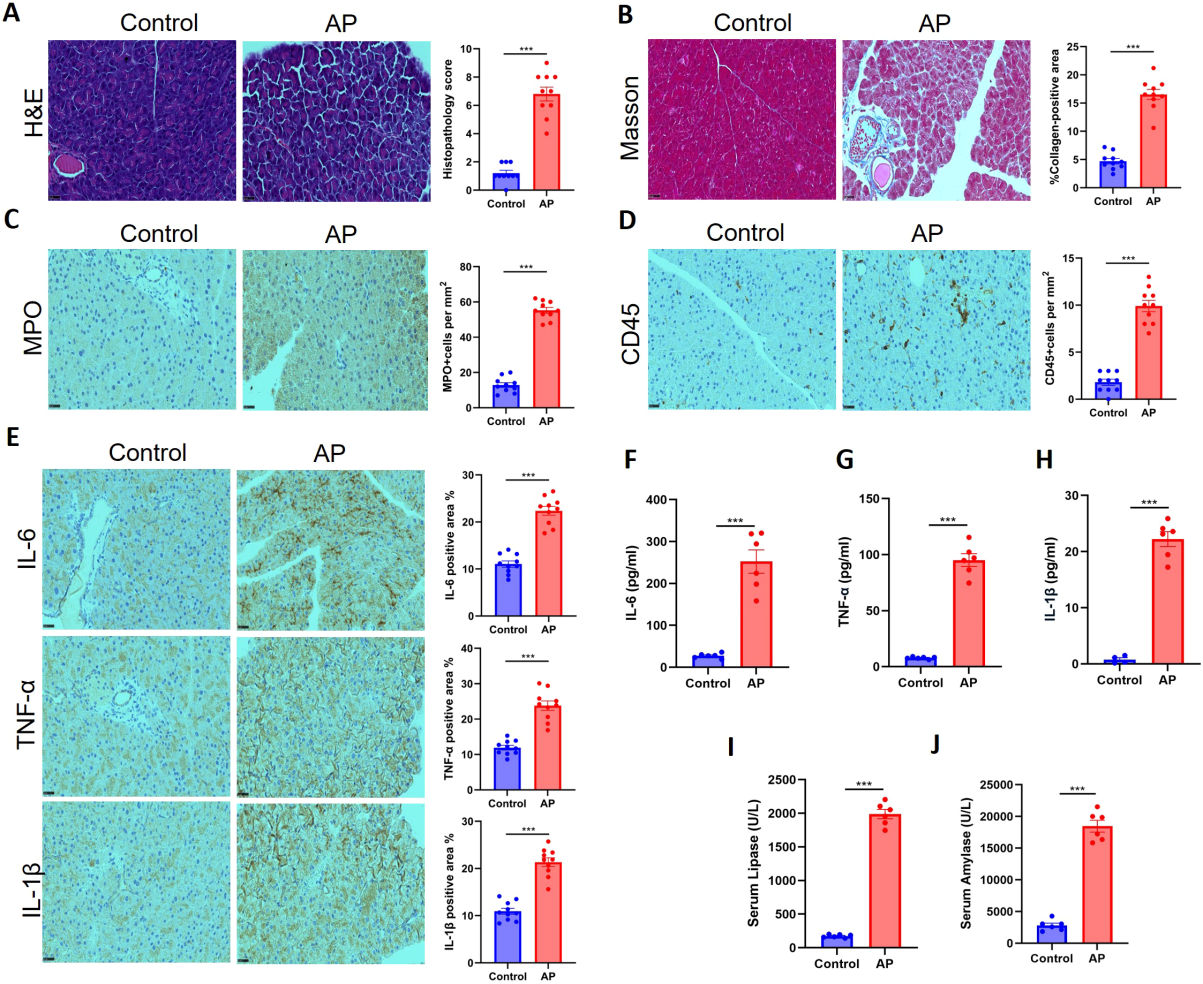
Cerulein successfully induces acute pancreatitis in SD rats. **(A)** Representative hematoxylin and eosin (H&E) staining of pancreatic tissue sections from saline-treated control and cerulein-induced acute pancreatitis (AP) model rats at 7 days post-induction. Scale bar, 100 μm. **(B)** Representative Masson’s trichrome staining showing collagen deposition (blue) in the pancreatic parenchyma. Scale bar, 100 μm. **(C, D)** Immunohistochemical (IHC) staining and quantification of MPO-positive neutrophils **(C)** and CD45-positive leukocytes **(D)** in pancreatic tissues. Scale bar, 50 μm. Quantification is presented as positive cells per high-power field (HPF). **(E)** Semi-quantitative H-score analysis of IHC staining for pro-inflammatory cytokines IL-6, TNF-α, and IL-1β in pancreatic tissue sections. **(F–H)** Serum levels of IL-6 **(F)**, TNF-α **(G)**, and IL-1β **(H)** measured by enzyme-linked immunosorbent assay (ELISA). **(I, J)** Serum amylase **(I)** and lipase **(J)** activities in control and AP groups. All data are presented as mean ± SEM (n=6 biologically independent animals per group). Statistical significance was determined by unpaired two-tailed Student’s *t*-test. ***p < 0.001 versus control group.

H&E staining confirmed the successful induction of acute pancreatitis (AP) in the cerulein-treated group, as evidenced by characteristic histopathological alterations including marked interstitial edema, extensive inflammatory cell infiltration, acinar cell vacuolization, and focal necrosis, in stark contrast to the normal architecture observed in control tissues (**Figure 1A**). Notably, Masson’s trichrome staining revealed that this acute injury was accompanied by significant collagen deposition and early fibrotic alterations within the pancreatic parenchyma at the 7-day time point, suggesting an active pro-fibrotic response (**Figure 1B**). To further characterize the inflammatory infiltrate, immunohistochemistry was performed, demonstrating a substantial influx of MPO-positive neutrophils and a generalized increase in CD45-positive leukocytes in the cerulein-AP group (**Figures 1C, D**). This robust cellular inflammation was paralleled by a marked upregulation in the local expression of key pro-inflammatory cytokines (IL-6, TNF-α, and IL-1β) within the pancreatic tissue, as confirmed by semi-quantitative analysis of immunoreactivity (**Figure 1E**). Corroborating these local findings, systemic analysis of serum samples via ELISA showed significantly elevated levels of these same cytokines (IL-6, TNF-α, IL-1β) in the model group (**Figures 1F–H**). Finally, the classic biochemical hallmarks of pancreatic injury were confirmed, with serum levels of lipase and amylase being significantly elevated in cerulein-treated rats, providing definitive serological evidence of successful AP induction (**Figures 1I, J**).

### 3.2 Single-cell transcriptomic profiling identifies MXD3 as a key regulator of epithelial remodeling in acute pancreatitis

**Figure 2:**
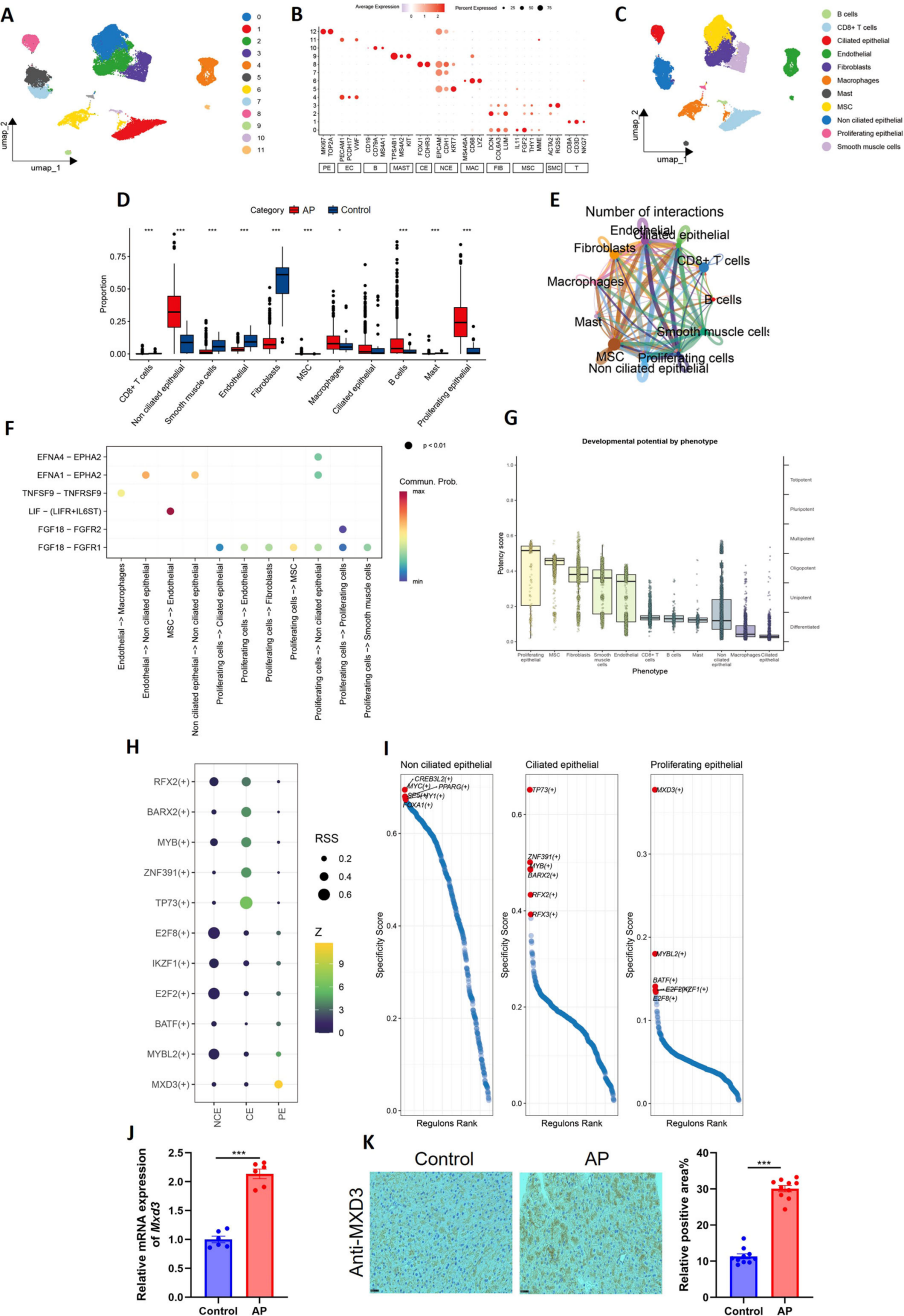
Single-cell transcriptomic profiling identifies MXD3 as a key regulator of epithelial remodeling in acute pancreatitis. **(A)** Uniform Manifold Approximation and Projection (UMAP) plot visualizing 38,521 single cells from pancreatic tissues of saline-treated control and cerulein-induced AP rats (n=3 per group). Cells are colored by cluster identity, revealing 11 distinct populations. **(B)** Dot plot showing the expression of canonical marker genes used for cell type annotation. Dot size represents the percentage of cells expressing the gene, and color intensity indicates average expression level. **(C)** Feature plots displaying the expression of representative marker genes for each major cell type. **(D)** Bar plot showing the relative proportion of each cell type in control versus AP groups. Statistical comparison for non-ciliated and proliferative epithelial populations was performed using a two-proportions z-test. **(E)** Cell-cell interaction network inferred from scRNA-seq data using CellPhoneDB. Edge width indicates the number of significant ligand-receptor pairs between cell types. **(F)** Heatmap of selected ligand-receptor pairs showing communication strength between proliferative epithelial cells and other cell populations. **(G)** Phenotypic trajectory scoring assigning pathogenic potential to each epithelial subpopulation (CE: ciliated epithelial; NCE: non-ciliated epithelial; PE: proliferative epithelial). **(H)** Heatmap of pySCENIC-derived regulon activity scores across the three epithelial subpopulations. Selected transcription factors (TFs) with differential activity are shown. **(I)** Venn diagram intersecting BEAM (branched expression analysis modeling)-derived trajectory-dependent genes with regulons from **(H)** to identify core TFs governing epithelial plasticity. Six TFs (ANKH, CEP126, WDR35, ORC6, EZH2, MXD3) were identified. **(J)** Quantitative PCR (qPCR) validation of MXD3 mRNA expression in pancreatic tissues from control and AP rats (n=6 per group). Data are normalized to GAPDH and presented as mean ± SEM. **(K)** Representative IHC staining and quantification of MXD3 protein expression in pancreatic tissue sections from control and AP rats. Scale bar, 50 μm. H-score quantification is shown on the right (n=6 per group). Data are mean ± SEM. For (D, J, K), statistical significance was determined by unpaired two-tailed Student’s *t*-test. ***p < 0.001.

To systematically characterize the cellular heterogeneity and transcriptional dynamics underlying acute pancreatitis (AP), we performed single-cell RNA sequencing (scRNA-seq) on pancreatic tissues from cerulein-induced AP rats and saline-treated controls (n=3 per group). Unbiased clustering of 38,521 high-quality cells identified 11 distinct cellular populations (**Figure 2A**), which were annotated based on canonical marker gene expression (**Figures 2B, C**): B cells (CD19, CD79A, MS4A1), CD8^+^ T cells (CD8A, CD3D, NKG7), ciliated epithelial cells (FOXJ1, CDHR3), endothelial cells (PECAM1, PCDH17, VWF), fibroblasts (DCN, COL6A3, LUM), macrophages (MS4A6A, CD68, LYZ), mast cells (TPSAB1, MS4A2, KIT), mesenchymal stem cells (IL11, FGF2, THY1, MME), non-ciliated epithelial cells (EPCAM, CDH1, KRT7), proliferative epithelial cells (MKI67, TOP2A), and smooth muscle cells (ACTA2, RGS5).

Comparative analysis of cellular composition between control and AP groups revealed substantial remodeling of the pancreatic cellular landscape. Notably, we observed a significant reduction in the non-ciliated epithelial cell population (p < 0.001), accompanied by a marked expansion of proliferative epithelial cells (p < 0.001) in AP tissues (**Figure 2D**), suggesting injury-induced epithelial dedifferentiation and subsequent proliferative regeneration.

Given the prominence of this proliferative epithelial cluster, we next interrogated its intercellular communication networks. Cell-cell interaction analysis unveiled that the proliferative epithelial population engages in extensive crosstalk with multiple other cell types (**Figure 2E**). Subsequent ligand-receptor pairing analysis identified the FGF18-FGFR1 axis as a prominently activated signaling pathway between proliferative epithelial cells and various immune and stromal populations (**Figure 2F**), implicating this interaction in the modulation of the inflammatory microenvironment. Furthermore, phenotypic trajectory scoring based on established AP severity signatures assigned the highest pathogenic potential to the proliferative epithelial cluster (**Figure 2G**), underscoring its putative role as a key driver of disease progression.

To elucidate the transcriptional regulatory circuitry governing this pathogenic epithelial state transition, we employed pySCENIC (single-cell regulatory network inference and clustering) to reconstruct cell-type-specific regulon activities across the three epithelial subpopulations: ciliated (CE), non-ciliated (NCE), and proliferative epithelial (PE) cells (**Figure 2H**). Intersecting these regulons with BEAM (branched expression analysis modeling)-derived trajectory-dependent genes (**Figure 2I**) pinpointed six core transcription factors (TFs) dynamically regulating epithelial plasticity: ANKH, CEP126, WDR35, ORC6, EZH2, and MXD3. Among these, MXD3 emerged as the most strikingly and specifically upregulated TF within the proliferative epithelial cluster, positioning it as a putative master transcriptional driver of AP-associated epithelial reprogramming.

The functional relevance of MXD3 upregulation was further corroborated in an independent cohort of cerulein-induced AP rats. Quantitative PCR (qPCR) and immunohistochemical (IHC) analyses confirmed significant elevation of both MXD3 mRNA and protein levels in pancreatic tissues from AP animals compared to saline-treated controls (**Figures 2J, K**), validating our scRNA-seq findings at the tissue level.

Collectively, these multifaceted data nominate MXD3 as a critical transcriptional orchestrator of the epithelial proliferative response and pathological remodeling characteristic of acute pancreatic injury, positioning it as a potential candidate for therapeutic intervention.

### 3.3 Epithelial-specific deletion of MXD3 attenuates cerulein-induced acute pancreatitis in rats

To investigate the pathogenic role of MXD3 specifically within pancreatic epithelial cells *in vivo*, we generated a conditional knockout (CKO) rat model by crossing *MXD3flox/flox rats with rats expressing Cre recombinase under the control of the Ck19 promoter, which directs specific expression in pancreatic ductal epithelial cells. This yielded experimental MXD3flox/flox; Ck19-Cre (hereafter referred to as MXD3 CKO) rats, with their MXD3flox/flox; Cre- littermates serving as wild-type (WT) controls.

**Figure 3:**
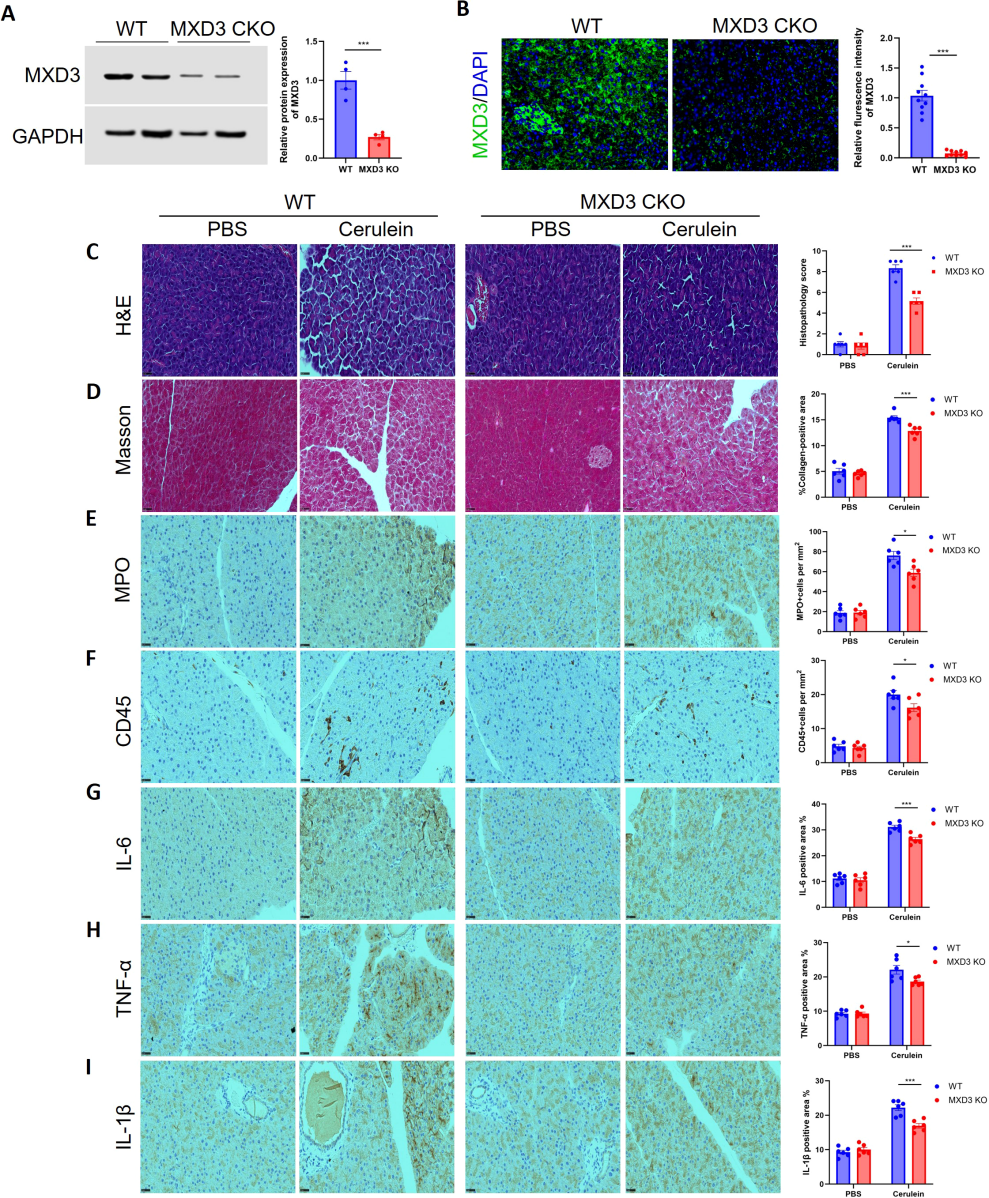
Epithelial-specific deletion of MXD3 attenuates cerulein-induced acute pancreatitis in rats. **(A)** Western blot analysis of MXD3 protein expression in pancreatic tissue lysates from wild-type (WT) and MXD3 conditional knockout (CKO) rats. β-actin served as a loading control. (n=4 biologically independent animals per group). Data are mean ± SEM. **(B)** Immunofluorescence co-staining for the epithelial marker CK19 (green) and MXD3 (red) in pancreatic tissue sections from WT and MXD3 CKO rats. Nuclei were counterstained with DAPI (blue). Scale bar, 20 μm. (n=4 animals per group, 5 fields per animal). Data are mean ± SEM. **(C)** Representative H&E staining of pancreatic sections from WT and MXD3 CKO rats treated with PBS (vehicle) or cerulein. Scale bar, 100 μm. **(D)** Representative Masson’s trichrome staining and quantification of fibrotic area (collagen deposition) in pancreatic sections from the indicated groups (n=6 per group). Scale bar, 100 μm. **(E, F)** Representative IHC staining and quantification of MPO-positive neutrophils **(E)** and CD45-positive leukocytes **(F)** in pancreatic tissues from WT and MXD3 CKO rats after cerulein treatment. Scale bar, 50 μm. Quantification is presented as positive cells per HPF (n=6 per group). Data are mean ± SEM. **(G–I)** Representative IHC staining and H-score quantification of IL-6 **(G)**, TNF-α **(H)**, and IL-1β **(I)** expression in pancreatic tissues from cerulein-treated WT and MXD3 CKO rats (n=6 per group). Scale bar, 50 μm. Data are mean ± SEM. For **(A-I)**, statistical significance was determined by unpaired two-tailed Student’s *t*-test **(A, B, E–I)** or one-way ANOVA with Tukey’s *post-hoc* test **(C, D)**. *p < 0.05, ***p < 0.001.

The efficiency and specificity of the knockout were first validated. Western blot analysis of pancreatic tissue extracts revealed a significant reduction of MXD3 protein in MXD3 CKO rats compared to WT controls (**Figure 3A**). This finding was corroborated by immunofluorescence staining, which confirmed the marked ablation of MXD3 expression specifically within the pancreatic ductal epithelium of MXD3 CKO rats (**Figure 3B**).

We next subjected both WT and MXD3 CKO rats to cerulein-induced acute pancreatitis (AP) or PBS (vehicle) control treatment to assess the functional consequence of MXD3 loss. Under baseline conditions (PBS treatment), pancreatic tissues from WT and MXD3 CKO rats were histologically indistinguishable. However, following cerulein challenge, the MXD3 CKO rats exhibited a pronounced attenuation of pancreatic injury. Quantitative analysis of H&E-stained sections demonstrated a significantly lower histological score in MXD3 CKO rats compared to WT controls, indicating reduced severity of tissue damage, edema, and inflammatory cell infiltration (**Figure 3C**). Concordantly, Masson’s trichrome staining revealed a substantial decrease in collagen deposition in MXD3 CKO rats, suggesting that the deletion of MXD3 mitigated cerulein-triggered fibrotic responses (**Figure 3D**).

The protective effect of MXD3 knockout was further supported by a significant reduction in the pancreatic inflammatory infiltrate. Immunohistochemistry for myeloperoxidase (MPO) showed fewer infiltrating neutrophils in MXD3 CKO rats after cerulein administration compared to WT rats (**Figure 3E**). Similarly, staining for CD45, a pan-leukocyte marker, indicated a globally diminished leukocyte influx in the knockout group (**Figure 3F**). Finally, the expression of key pro-inflammatory cytokines (IL-6, TNF-α, and IL-1β) within the pancreatic tissue was significantly downregulated in cerulein-treated MXD3 CKO rats relative to their WT counterparts (**Figures 3G–I**).

Collectively, these data demonstrate that specific ablation of MXD3 in pancreatic ductal epithelial cells confers a protective phenotype against cerulein-induced acute pancreatitis, manifesting as diminished histopathological damage, reduced fibrosis, attenuated neutrophil infiltration, and a blunted pro-inflammatory cytokine response.

### 3.4 MXD3-mediated epithelial remodeling in acute pancreatitis is linked to Wnt/β-catenin pathway activation

**Figure 4:**
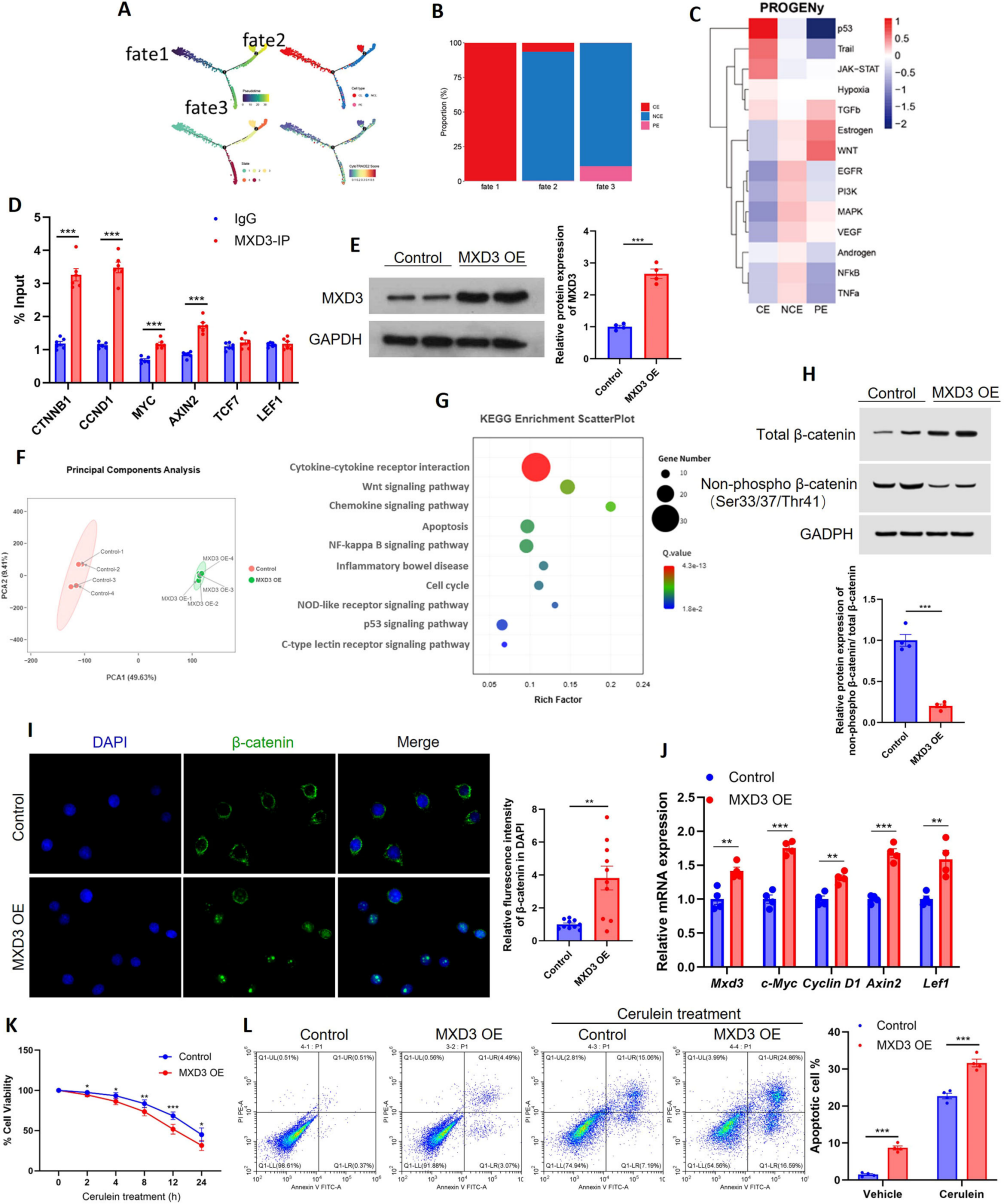
MXD3-mediated epithelial remodeling in acute pancreatitis is linked to Wnt/β-catenin pathway activation. **(A)** Pseudotime trajectory analysis of epithelial subpopulations (CE, NCE, PE) projected onto UMAP. Cells are colored by pseudotime, illustrating a differentiation path from ciliated (CE) through non-ciliated (NCE) to proliferative (PE) fate. **(B)** Bar plot showing the distribution of cells along the three identified fates (Fate 1: CE, Fate 2: NCE, Fate 3: PE). **(C)** PROGENy pathway activity analysis across epithelial subpopulations. Heatmap shows the relative activity of canonical signaling pathways. The Wnt pathway is specifically and significantly activated in the PE cluster. **(D)** Chromatin immunoprecipitation followed by qPCR (ChIP-qPCR) analysis of MXD3 occupancy at the promoter regions of indicated Wnt pathway genes (CTNNB1, CCND1, MYC, AXIN2, TCF7, LEF1) in MXD3-overexpressing HPDE cells. Enrichment is presented as percentage of input DNA relative to IgG control. **(E)** Western blot confirmation of MXD3 overexpression (MXD3 OE) in HPDE cells compared to empty vector control (Vector). β-actin served as loading control. **(F)** Principal component analysis (PCA) plot of RNA-seq data from Vector and MXD3 OE HPDE cells. **(G)** KEGG pathway enrichment analysis of differentially expressed genes (DEGs) between MXD3 OE and Vector groups. Top 10 enriched pathways are shown. **(H)** Western blot analysis of total β-catenin and non-phosphorylated (active) β-catenin (Ser33/37/Thr41) in Vector and MXD3 OE HPDE cells. **(I)** Immunofluorescence staining of β-catenin (green) in Vector and MXD3 OE HPDE cells. Nuclei were counterstained with DAPI (blue). Scale bar, 20 μm. **(J)** qPCR analysis of Wnt/β-catenin target gene mRNA expression (MYC, CCND1, AXIN2, LEF1) in Vector and MXD3 OE HPDE cells. **(K)** Cell viability assessed by MTT assay in Vector and MXD3 OE HPDE cells treated with cerulein (15 nM) for the indicated time points (0–24 h). Data are presented as percentage of untreated control (0 h). **(L)** Flow cytometric analysis of apoptosis (Annexin V/PI staining) in Vector and MXD3 OE HPDE cells under basal conditions or after stimulation with cerulein (15 nM, 24 h). Representative flow plots are shown on the left; quantification of apoptotic cells (Annexin V-positive) is shown on the right. Data are mean ± SEM (n=4 independent biological replicates). For **(D, H–L)**, statistical significance was determined by unpaired two-tailed Student’s *t*-test **(D, H–J)** or two-way ANOVA with Sidak’s *post-hoc* test **(K)** or one-way ANOVA with Tukey’s *post-hoc* test **(L)**. *p < 0.05, **p < 0.01, ***p < 0.001.

To elucidate the molecular mechanism by which MXD3 orchestrates epithelial pathology in acute pancreatitis (AP), we first leveraged our single-cell RNA sequencing dataset. Pseudotime trajectory analysis of the epithelial subpopulations delineated a progressive dedifferentiation path culminating in the pathogenic proliferative state (**Figure 4A**). This trajectory originated from ciliated epithelial cells (Fate 1), transitioned through a non-ciliated intermediate state (Fate 2), and converged on the proliferative epithelial fate (Fate 3), which we had previously identified as the most pathologically aggressive population (**Figure 4B**). To identify signaling pathways activated during the acquisition of this invasive phenotype, we performed quantitative pathway activity inference using PROGENy. This analysis revealed a specific and pronounced activation of the Wnt signaling pathway within the proliferative epithelial cluster (**Figure 4C**), suggesting its potential role in driving this detrimental cellular state. To determine whether MXD3 directly regulates the transcription of Wnt pathway components, we performed chromatin immunoprecipitation followed by qPCR (ChIP-qPCR). These experiments demonstrated significant enrichment of MXD3 binding at the promoter regions of key Wnt pathway genes, including CTNNB1, CCND1, MYC, and AXIN2 (**Figure 4D**), providing direct evidence of MXD3’s role as a transcriptional regulator of this pathway.

Building on these bioinformatic predictions, we hypothesized that MXD3 exerts its pro-pathogenic effects through the modulation of Wnt/β-catenin signaling. We first tested this hypothesis in human pancreatic ductal epithelial (HPDE) cells. Following successful overexpression of MXD3 (confirmed by Western blot, **Figure 4E**), we performed bulk RNA-sequencing (RNA-seq). Principal component analysis (PCA) revealed a clear transcriptional separation between MXD3-overexpressing (MXD3 OE) and control cells (**Figure 4F**). KEGG pathway enrichment analysis of the differentially expressed genes (DEGs) confirmed the significant enrichment of the Wnt signaling pathway, alongside other AP-relevant pathways such as apoptosis and NF-κB signaling (**Figure 4G**), indicating that MXD3 orchestrates a broader transcriptional network.

We next assessed the core hallmarks of canonical Wnt pathway activation: β-catenin stabilization and nuclear translocation. Western blot analysis demonstrated that MXD3 OE significantly increased the ratio of non-phosphorylated (active) β-catenin (at Ser33/37/Thr41) to total β-catenin, indicating inhibition of the β-catenin destruction complex and subsequent protein stabilization (**Figure 4H**). Furthermore, immunofluorescence staining confirmed a marked accumulation of β-catenin within the nucleus upon MXD3 overexpression (**Figure 4I**), providing gold-standard evidence for the activation of canonical Wnt signaling. Finally, to validate the functional output of this pathway activation, we examined the mRNA expression levels of key Wnt/β-catenin downstream target genes. Quantitative PCR (qPCR) results confirmed that MXD3 OE significantly upregulated the transcript levels of c-Myc, Cyclin D1, Axin2, and LEF1 (**Figure 4J**).

To extend these mechanistic findings to a disease-relevant context, we next investigated the functional consequences of MXD3 OE on HPDE cell fate under both basal and AP-like stress conditions. Cell viability, assessed by MTT assay over a time course of cerulein stimulation (0–24 h), revealed that MXD3 OE significantly exacerbated cerulein-induced cytotoxicity compared to control cells (**Figure 4K**). Concurrently, flow cytometric analysis of apoptosis (Annexin V/PI staining) demonstrated that MXD3 OE not only increased basal apoptotic rates but also significantly potentiated cerulein-induced apoptosis (**Figure 4L**). These results indicate that MXD3 overexpression sensitizes pancreatic epithelial cells to injury-induced cell death.

Collectively, these data establish a direct mechanistic link between MXD3 and the Wnt/β-catenin pathway. Our findings support a model in which MXD3 functions as an upstream transcriptional activator, driving the expression of pro-proliferative and pro-pathogenic genes in pancreatic epithelial cells, thereby contributing to disease progression in acute pancreatitis.

### 3.5 MXD3 exacerbates pancreatic epithelial cell injury in a Wnt/β catenin-dependent manner

To definitively establish whether the Wnt/β-catenin pathway serves as the critical downstream effector of MXD3 in pancreatic epithelial pathology, we employed a loss-of-function strategy using both pharmacological and genetic approaches. A four-group experimental design was implemented in HPDE cells: (1) empty vector control (Vector), (2) vector control challenged with TNF-α (Vector + TNF-α), (3) MXD3 overexpression challenged with TNF-α (MXD3 OE + TNF-α), and (4) MXD3 overexpression challenged with TNF-α in the presence of ICG-001 (MXD3 OE + TNF-α + ICG-001), a specific small-molecule inhibitor that disrupts β-catenin/TCF-mediated transcription.

**Figure 5:**
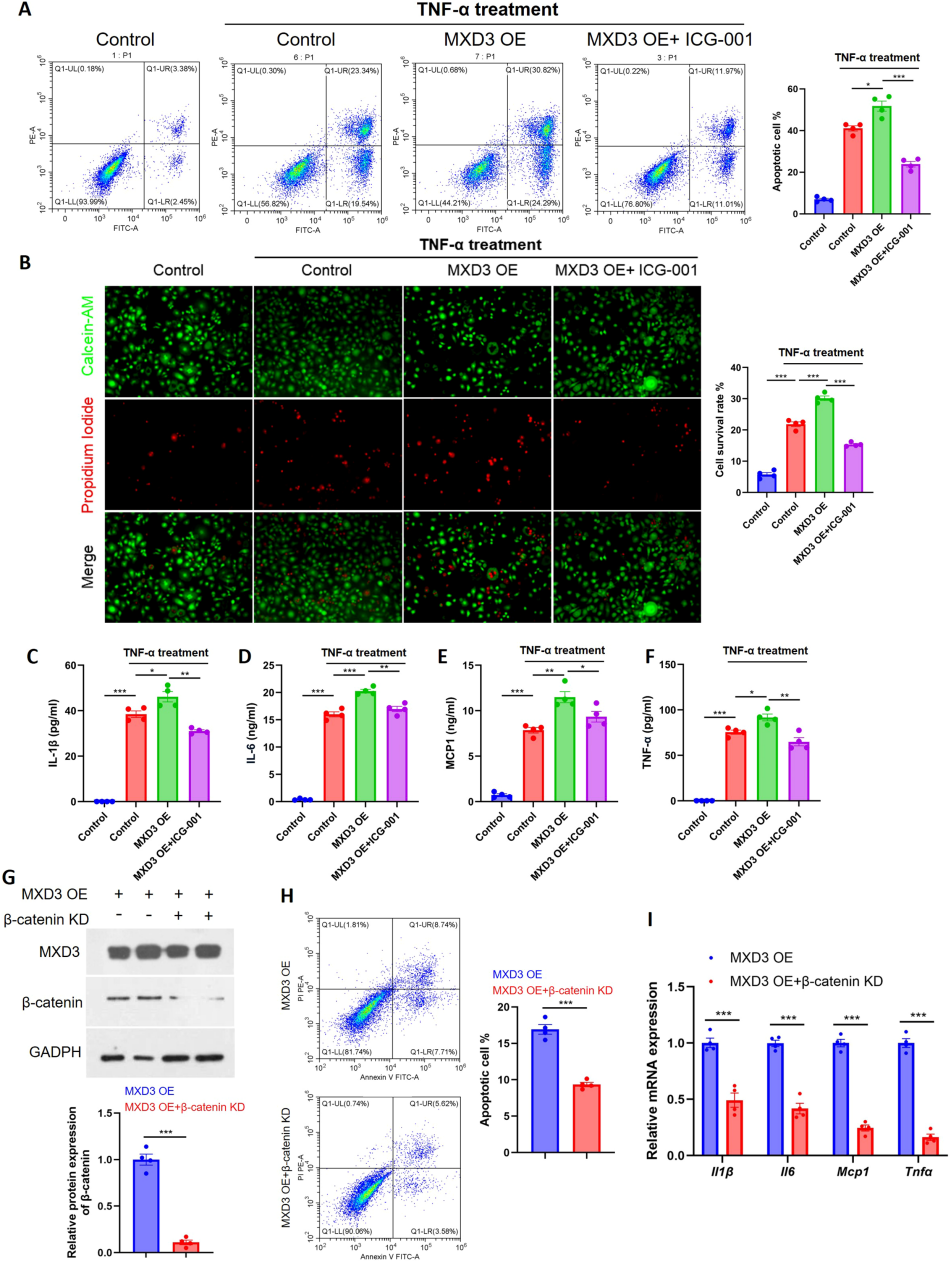
MXD3 exacerbates pancreatic epithelial cell injury in a Wnt/β-catenin-dependent manner. **(A)** Flow cytometric analysis of apoptosis (Annexin V/PI staining) in HPDE cells under the indicated conditions: Vector, Vector + TNF-α, MXD3 OE + TNF-α, and MXD3 OE + TNF-α + ICG-001 (10 μM). **(B)** Representative Calcein-AM (live, green) and PI (dead, red) staining of HPDE cells under the conditions described in **(A)**. Scale bar, 100 μm. Quantification of PI-positive (necrotic) cells is shown on the right. Data are mean ± SEM (n=4 independent biological replicates, >5 fields quantified per replicate). **(C–F)** ELISA quantification of IL-1β **(C)**, IL-6 **(D)**, MCP-1 **(E)**, and TNF-α **(F)** secretion into the culture supernatant of HPDE cells under the indicated conditions. Data are mean ± SEM (n=4 independent biological replicates). **(G)** Western blot confirmation of MXD3 overexpression and β-catenin knockdown (si-β-catenin) efficiency in HPDE cells. **(H)** Flow cytometric analysis of apoptosis (Annexin V/PI staining) in HPDE cells under the conditions shown in **(G)**. Quantification of apoptotic cells (Annexin V-positive) is presented. Data are mean ± SEM (n=4 independent biological replicates). **(I)** qPCR analysis of inflammatory cytokine mRNA expression (IL1B, IL6, CCL2, TNF) in HPDE cells under the conditions shown in **(G)**. Data are normalized to GAPDH and presented as mean ± SEM (n=4 independent biological replicates). For (A-F, H, I), statistical significance was determined by one-way ANOVA with Tukey’s *post-hoc* test. *p < 0.05, **p < 0.01, ***p < 0.001.

Flow cytometric analysis of apoptosis (Annexin V/PI staining) revealed that MXD3 overexpression significantly potentiated TNF-α-induced apoptosis compared to vector control cells. Strikingly, this pro-apoptotic effect was markedly attenuated upon co-treatment with ICG-001, indicating its dependence on intact Wnt/β-catenin transcriptional activity (**Figure 5A**). We next evaluated necrotic cell death using Calcein-AM/PI live/dead imaging. Consistent with the apoptosis data, MXD3 overexpression substantially enhanced TNF-α-induced necrosis, and this effect was again significantly rescued by Wnt pathway inhibition (**Figure 5B**).

Furthermore, we investigated the impact of MXD3 on the inflammatory secretome. ELISA analysis of cell culture supernatants demonstrated that MXD3 overexpression led to a significant increase in the secretion of key pro-inflammatory cytokines, including IL-1β, IL-6, MCP-1, and TNF-α. Administration of ICG-001 effectively reduced the levels of these cytokines by approximately 25–30%, returning them towards baseline (**Figures 5C-F**), suggesting that a substantial component of MXD3’s pro-inflammatory effect is mediated through Wnt/β-catenin signaling.

Given the potential for off-target effects inherent to pharmacological inhibitors, we sought to genetically validate our findings by performing β-catenin knockdown in the context of MXD3 overexpression. Western blot analysis confirmed efficient MXD3 overexpression and successful β-catenin silencing (**Figure 5G**). Remarkably, flow cytometric analysis revealed that β-catenin knockdown significantly abrogated the pro-apoptotic effect conferred by MXD3 OE (**Figure 5H**). Concordantly, qPCR analysis demonstrated that β-catenin silencing also reversed the MXD3-induced upregulation of inflammatory cytokine transcripts, including IL1B, IL6, CCL2 (MCP-1), and TNF (**Figure 5I**). This genetic rescue experiment provides compelling evidence for the specificity and necessity of β-catenin in mediating MXD3’s pathogenic effects.

Collectively, these complementary pharmacological and genetic rescue experiments establish that MXD3’s pathogenic role in driving pancreatic epithelial cell injury—manifested as enhanced apoptosis, necrosis, and inflammatory amplification—is primarily mediated through the activation of canonical Wnt/β-catenin signaling.

## 4 Discussion

Acute pancreatitis (AP) represents a complex inflammatory disorder with significant morbidity and mortality, yet its underlying molecular mechanisms remain incompletely elucidated. While initial trypsinogen activation in acinar cells is recognized as a triggering event (Wang et al., 2009; Watanabe et al., 2017), the subsequent transcriptional reprogramming that drives disease progression requires further investigation. Our integrated multi-omics study identifies MXD3 as a previously uncharacterized master regulator of epithelial pathogenesis in AP, establishing its crucial function through Wnt/β-catenin pathway activation.

The power of single-cell transcriptomics allowed us to delineate the cellular landscape of cerulein-induced AP with unprecedented resolution. We identified a distinct dedifferentiation trajectory wherein epithelial cells transition from a ciliated to a proliferative phenotype, consistent with emerging paradigms of epithelial plasticity in pancreatic pathology (Pinho et al., 2011; Schmidtlein et al., 2021; Peura et al., 2025). This cellular transition mirrors findings in pancreatic cancer precursors (Schmidtlein et al., 2021), where similar differentiation states are associated with disease progression. The pronounced upregulation of MXD3 within the pathogenic proliferative cluster was particularly striking, suggesting its potential role in driving this detrimental transformation. This observation gains significance from recent studies showing that transcription factors regulating cell cycle progression can profoundly influence pancreatic injury responses (Sojoodi et al., 2016; Zhou et al., 2024).

The functional validation of MXD3’s role through tissue-specific knockout models provided compelling evidence for its central position in AP pathogenesis. The remarkable protection observed in MXD3-CKO rats—manifested through reduced histological damage, diminished fibrosis, and attenuated inflammation—underscores the therapeutic potential of targeting this pathway. Our findings align with growing evidence that the ductal epithelium serves as an active participant in pancreatic inflammation rather than merely a passive victim (Lee and Bar-Sagi, 2010; Fernández et al., 2024). The reduction in neutrophil infiltration and pro-inflammatory cytokine expression in knockout animals particularly emphasizes MXD3’s role in modulating the immune microenvironment, reminiscent of mechanisms described in pancreatic ductal adenocarcinoma progression. To further explore how epithelial MXD3 deletion leads to reduced inflammation, we performed ligand-receptor interaction analysis using our scRNA-seq data. This revealed a significant enrichment of the FGF18-FGFR1 signaling axis between the proliferative epithelial cluster and various immune cells (**Figures 2E, F**), a pathway previously implicated in inflammatory regulation. While these findings provide bioinformatic evidence for direct epithelial-immune crosstalk, we acknowledge that the attenuated inflammation observed in MXD3-CKO rats likely results from a combination of direct effects (altered epithelial-derived signals) and indirect effects secondary to reduced epithelial cell injury and subsequent damage-associated molecular pattern release. This complex, bidirectional relationship between epithelial cells and immune cells warrants further investigation.

Mechanistically, we uncovered that MXD3 exerts its pathogenic effects primarily through Wnt/β-catenin signaling activation. Our integrated analysis, combining RNA-seq (**Figures 4F, G**) with functional validation, confirms Wnt/β-catenin as a central downstream pathway. However, the enrichment of other pathways, such as NF-κB signaling and apoptosis, suggests that MXD3 may orchestrate a broader transcriptional network contributing to AP pathology. Our demonstration of β-catenin stabilization, nuclear translocation, and subsequent target gene upregulation provides a comprehensive picture of pathway engagement. To establish direct transcriptional regulation, we performed ChIP-qPCR assays, which demonstrated significant enrichment of MXD3 binding at the promoter regions of key Wnt pathway genes, including CTNNB1, CCND1, MYC, and AXIN2 (**Figure 4D**). This provides direct evidence that MXD3 activates the Wnt pathway through promoter binding. This connection is particularly intriguing given Wnt signaling’s established roles in development and cancer (Nusse and Clevers, 2017; Taciak et al., 2018; Yu et al., 2021), yet its regulation in inflammatory pancreatic conditions remains poorly understood. The specificity of this interaction was confirmed through multiple experimental approaches. We demonstrated that both pharmacological inhibition (ICG-001) and genetic knockdown of β-catenin (**Figures 5H, I**) could significantly reverse MXD3-mediated apoptosis and pro-inflammatory effects, further strengthening the causal relationship. This mechanistic insight extends recent work suggesting Wnt pathway involvement in AP (Huang et al., 2022; Sun et al., 2023), while providing the novel finding that MXD3 serves as its crucial upstream regulator.

The rescue experiments using ICG-001 offered particularly convincing evidence for the pathway’s necessity in MXD3-mediated pathology. The reversal of apoptotic, necrotic, and inflammatory responses upon β-catenin inhibition strongly supports our model of a linear MXD3-Wnt/β-catenin axis driving epithelial dysfunction.

Our study does have limitations that warrant consideration. First, while we established the MXD3-Wnt/β-catenin axis as crucial, the precise upstream molecular mechanisms governing MXD3 upregulation during AP remain unexplored. Potential regulators, such as inflammatory signaling pathways (e.g., NF-κB or STAT3) or cellular stress responses, should be investigated in future studies. Second, although our ChIP-qPCR data confirm MXD3 binding to Wnt pathway gene promoters, the potential involvement of other signaling pathways downstream of MXD3 cannot be completely excluded and merits further exploration, particularly given our pathway analysis results (**Figure 4G**). Third, the sample sizes for our *in vivo* experiments (n=6 per group) and scRNA-seq (n=3) were relatively modest, and power calculations were not performed prior to the study. This, along with the lack of biological replication for the scRNA-seq experiment, may limit the statistical robustness and confidence in some downstream analyses, such as trajectory inference. These factors were partly due to budgetary constraints and have been acknowledged as limitations. Fourth, our *in vitro* experiments focused on MXD3 overexpression; complementary MXD3 knockdown studies in epithelial cells would provide a more complete picture of its loss-of-function effects. Fifth, the translational potential of targeting MXD3 in human AP requires validation. Due to institutional limitations and the current lack of publicly available human AP transcriptomic datasets with detailed clinical information, we were unable to correlate MXD3 expression with disease severity in human samples. Future collaborative efforts to obtain and validate MXD3 expression in human AP tissues are necessary to confirm the clinical relevance of our findings.

In conclusion, our work establishes MXD3 as a pivotal regulator of epithelial pathogenesis in acute pancreatitis, functioning through Wnt/β-catenin pathway activation. These findings significantly advance our understanding of AP pathophysiology while revealing a promising new therapeutic target. Future studies investigating MXD3 inhibition strategies, the upstream signals that control its expression and their effects on disease progression could open new avenues for clinical intervention in this challenging condition.

## Data Availability

The data presented in the study are deposited in the Gene Expression Omnibus (GEO) repository, accession number GSE328424.

## References

[B1] Boxhoorn L. Voermans R. P. Bouwense S. A. Bruno M. J. Verdonk R. C. Boermeester M. A. . (2020). Acute pancreatitis. Lancet 396, 726–734. doi: 10.1016/S0140-6736(20)31310-6. PMID: 32891214

[B2] Chi H. C. Tsai C. Y. Wang C. S. Yang H. Y. Lo C. H. Wang W. J. . (2020). DOCK6 promotes chemo- and radioresistance of gastric cancer by modulating WNT/β-catenin signaling and cancer stem cell traits. Oncogene 39, 5933–5949. doi: 10.1038/s41388-020-01390-0. PMID: 32753649

[B3] Del Poggetto E. Ho I. L. Balestrieri C. Yen E. Y. Zhang S. Citron F. . (2021). Epithelial memory of inflammation limits tissue damage while promoting pancreatic tumorigenesis. Science 373, eabj0486. doi: 10.1126/science.abj0486. PMID: 34529467 PMC9733946

[B4] Fernández Á. Casamitjana J. Holguín-Horcajo A. Coolens K. Mularoni L. Guo L. . (2024). A single-cell atlas of the murine pancreatic ductal tree identifies novel cell populations with potential implications in pancreas regeneration and exocrine pathogenesis. Gastroenterology 167, 944–960.e15. doi: 10.1053/j.gastro.2024.06.008. PMID: 38908487

[B5] Gaowa A. Leangpanich S. Park E. J. Kawamoto E. Shimaoka M. (2024). Irisin promotes intestinal epithelial cell proliferation via Wnt/β-catenin and focal adhesion kinase signaling pathways. Sci. Rep. 14, 25702. doi: 10.1038/s41598-024-76658-6. PMID: 39465344 PMC11514181

[B6] Guo K. Zhao Y. Cao Y. Li Y. Yang M. Tian Y. . (2023). Exploring the key genetic association between chronic pancreatitis and pancreatic ductal adenocarcinoma through integrated bioinformatics. Front. Genet. 14. doi: 10.3389/fgene.2023.1115660. PMID: 37501719 PMC10369079

[B7] Huang H. Chen W. Lu J. Zhang S. Xiang X. Wang X. . (2022). Circ_0000284 promoted acute pancreatitis progression through the regulation of miR-10a-5p/Wnt/β-catenin pathway. Chem. Biodivers. 19, e202101006. doi: 10.1002/cbdv.202101006. PMID: 35581162

[B8] Lee K. E. Bar-Sagi D. (2010). Oncogenic KRas suppresses inflammation-associated senescence of pancreatic ductal cells. Cancer Cell. 18, 448–458. doi: 10.1016/j.ccr.2010.10.020. PMID: 21075310 PMC3397918

[B9] Li Y. Liu C. Zhang X. Huang X. Liang S. Xing F. . (2022). CCT5 induces epithelial-mesenchymal transition to promote gastric cancer lymph node metastasis by activating the Wnt/β-catenin signalling pathway. Br. J. Cancer 126, 1684–1694. doi: 10.1038/s41416-022-01747-0. PMID: 35194191 PMC9174209

[B10] Mederos M. A. Reber H. A. Girgis M. D. (2021). Acute pancreatitis: A review. JAMA 325, 382–390. doi: 10.1001/jama.2020.20317. PMID: 33496779

[B11] Melzer M. K. Schirge S. Gout J. Arnold F. Srinivasan D. Burtscher I. . (2023). TBX3 is dynamically expressed in pancreatic organogenesis and fine-tunes regeneration. BMC Biol. 21, 55. doi: 10.1186/s12915-023-01553-x. PMID: 36941669 PMC10029195

[B12] Nusse R. Clevers H. (2017). Wnt/β-catenin signaling, disease, and emerging therapeutic modalities. Cell 169, 985–999. doi: 10.1016/j.cell.2017.05.016. PMID: 28575679

[B13] Peura J. Johnson C. Pitarresi J. R. (2025). To EMT or not to EMT: Ablation of mesenchymal tumor cell lineages reveals the essential role of EMT in pancreatic cancer initiation and evolution. Cancer Res. 85, 2146–2148. doi: 10.1158/0008-5472.CAN-25-1443. PMID: 40198902 PMC12878739

[B14] Pinho A. V. Rooman I. Reichert M. De Medts N. Bouwens L. Rustgi A. K. . (2011). Adult pancreatic acinar cells dedifferentiate to an embryonic progenitor phenotype with concomitant activation of a senescence programme that is present in chronic pancreatitis. Gut 60, 958–966. doi: 10.1136/gut.2010.225920. PMID: 21193456

[B15] Ren S. Song L. N. Zhao R. Tian Y. Wang Z. Q. (2025). Serum exosomal hsa-let-7f-5p: A potential diagnostic biomarker for metastatic pancreatic cancer detection. World J. Gastroenterol. 31, 109500. doi: 10.3748/wjg.v31.i26.109500. PMID: 40678708 PMC12264843

[B16] Schmidtlein P. M. Volz C. Braun R. Thürling I. Lapshyna O. Wellner U. F. . (2021). A comparative endocrine trans-differentiation approach to pancreatic ductal adenocarcinoma cells with different EMT phenotypes identifies quasi-mesenchymal tumor cells as those with highest plasticity. Cancers 13, 4663. doi: 10.3390/cancers13184663. PMID: 34572891 PMC8466512

[B17] Sojoodi M. Stradiot L. Tanaka K. Heremans Y. Leuckx G. Besson V. . (2016). The zinc finger transcription factor PW1/PEG3 restrains murine beta cell cycling. Diabetologia 59, 1474–1479. doi: 10.1007/s00125-016-3954-z. PMID: 27130279 PMC4901110

[B18] Sun Y. Gu R. Shen Z. Ye Y. (2023). MALAT1 knockdown alleviates myocardial injury in mice with severe acute pancreatitis via the miR-374a/Sp1/Wnt/β-catenin pathway. Am. J. Trans. Res. 15, 3928–3941. PMC10331685 37434842

[B19] Szatmary P. Grammatikopoulos T. Cai W. Huang W. Mukherjee R. Halloran C. . (2022). Acute pancreatitis: Diagnosis and treatment. Drugs 82, 1251–1276. doi: 10.1007/s40265-022-01766-4. PMID: 36074322 PMC9454414

[B20] Taciak B. Pruszynska I. Kiraga L. Bialasek M. Krol M. (2018). Wnt signaling pathway in development and cancer. J. Physiol. Pharmacol. 69. doi: 10.26402/jpp.2018.2.07. PMID: 29980141

[B21] Trikudanathan G. Yazici C. Evans Phillips A. Forsmark C. E. (2024). Diagnosis and management of acute pancreatitis. Gastroenterology 167, 673–688. doi: 10.1053/j.gastro.2024.02.052. PMID: 38759844

[B22] Valverde-López F. Martínez-Cara J. G. Redondo-Cerezo E. (2022). Acute pancreatitis. Med. Clín. 158, 556–563. doi: 10.1016/j.medcli.2021.12.012. PMID: 35277268

[B23] Wang G. J. Gao C. F. Wei D. Wang C. Ding S. Q. (2009). Acute pancreatitis: Etiology and common pathogenesis. World J. Gastroenterol. 15, 1427–1430. doi: 10.3748/wjg.15.1427. PMID: 19322914 PMC2665136

[B24] Watanabe T. Kudo M. Strober W. (2017). Immunopathogenesis of pancreatitis. Mucosal Immunol. 10, 283–298. doi: 10.1038/mi.2016.101. PMID: 27848953

[B25] Wiley M. B. Mehrotra K. Bauer J. Yazici C. Bialkowska A. B. Jung B. (2023). Acute pancreatitis: Current clinical approaches, molecular pathophysiology, and potential therapeutics. Pancreas 52, e335–e343. doi: 10.1097/MPA.0000000000002259. PMID: 38127317 PMC11913250

[B26] Xu J. H. Ni C. Y. Zhuang Y. Y. Li L. Lin Y. Xia Z. S. . (2023). Acute pancreatitis in intraductal papillary mucinous neoplasm: A single-center retrospective cohort study with systematic review and meta-analysis. BMC Gastroenterol. 23, 424. doi: 10.1186/s12876-023-02972-4. PMID: 38041073 PMC10690977

[B27] Yang L. Ye F. Liu J. Klionsky D. J. Tang D. Kang R. (2023). Extracellular SQSTM1 exacerbates acute pancreatitis by activating autophagy-dependent ferroptosis. Autophagy 19, 1733–1744. doi: 10.1080/15548627.2022.2152209. PMID: 36426912 PMC10262765

[B28] Yu F. Yu C. Li F. Zuo Y. Wang Y. Yao L. . (2021). Wnt/β-catenin signaling in cancers and targeted therapies. Signal Transduct. Target. Ther. 6, 307. doi: 10.1038/s41392-021-00701-5. PMID: 34456337 PMC8403677

[B29] Zhou F. Li D. Liu C. Li C. Li K. Shi L. . (2024). m6A-activated BACH1 exacerbates ferroptosis by epigenetic suppression HSPB1 in severe acute pancreatitis. Drug Dev. Res. 85, e22256. doi: 10.1002/ddr.22256. PMID: 39285641

